# White Matter Atrophy and Cognitive Dysfunctions in Neuromyelitis Optica

**DOI:** 10.1371/journal.pone.0033878

**Published:** 2012-04-03

**Authors:** Frederic Blanc, Vincent Noblet, Barbara Jung, François Rousseau, Felix Renard, Bertrand Bourre, Nadine Longato, Nadjette Cremel, Laure Di Bitonto, Catherine Kleitz, Nicolas Collongues, Jack Foucher, Stephane Kremer, Jean-Paul Armspach, Jerome de Seze

**Affiliations:** 1 Neuropsychology Service, Department of Neurology, University Hospital of Strasbourg, Strasbourg, France; 2 LINC (Cognitive Neurosciences and Imagery Laboratory), UMR 7237, University of Strasbourg and CNRS, Strasbourg, France; 3 CMRR (Memory Resource and Research Centre), Department of Neurology, University Hospital of Strasbourg, Strasbourg, France; 4 LSIIT (Image Science, Computer Science and Remote Sensing Laboratory), UMR 7005, University of Strasbourg and CNRS, Strasbourg, France; 5 INSERM U666, University Hospital of Strasbourg, Strasbourg, France; 6 Neuroradiology service, University Hospital of Strasbourg, Strasbourg, France; Institute Biomedical Research August Pi Sunyer (IDIBAPS) - Hospital Clinic of Barcelona, Spain

## Abstract

Neuromyelitis optica (NMO) is an inflammatory disease of central nervous system characterized by optic neuritis and longitudinally extensive acute transverse myelitis. NMO patients have cognitive dysfunctions but other clinical symptoms of brain origin are rare. In the present study, we aimed to investigate cognitive functions and brain volume in NMO. The study population consisted of 28 patients with NMO and 28 healthy control subjects matched for age, sex and educational level. We applied a French translation of the Brief Repeatable Battery (BRB-N) to the NMO patients. Using SIENAx for global brain volume (Grey Matter, GM; White Matter, WM; and whole brain) and VBM for focal brain volume (GM and WM), NMO patients and controls were compared. Voxel-level correlations between diminished brain concentration and cognitive performance for each tests were performed. Focal and global brain volume of NMO patients with and without cognitive impairment were also compared. Fifteen NMO patients (54%) had cognitive impairment with memory, executive function, attention and speed of information processing deficits. Global and focal brain atrophy of WM but not Grey Matter (GM) was found in the NMO patients group. The focal WM atrophy included the optic chiasm, pons, cerebellum, the corpus callosum and parts of the frontal, temporal and parietal lobes, including superior longitudinal fascicle. Visual memory, verbal memory, speed of information processing, short-term memory and executive functions were correlated to focal WM volumes. The comparison of patients with, to patients without cognitive impairment showed a clear decrease of global and focal WM, including brainstem, corticospinal tracts, corpus callosum but also superior and inferior longitudinal fascicles. Cognitive impairment in NMO patients is correlated to the decreased of global and focal WM volume of the brain. Further studies are needed to better understand the precise origin of cognitive impairment in NMO patients, particularly in the WM.

## Introduction

Neuromyelitis optica (NMO) is a central nervous system (CNS) inflammatory disease characterized by optic neuritis (ON) and longitudinally extensive acute transverse myelitis (ATM). First described by Eugene Devic and others in the 19th century as a distinct disease, NMO or Devic’s disease was then classified as a subtype of multiple sclerosis (MS) [1]. However, the discovery of an autoantibody called NMO-IgG that targets aquaporin-4 (AQP4), indicates that NMO is clearly a different CNS pathology [2]. Patients with NMO have an extensive loss of AQP4 and a decreased astrocyte concentration in acute and chronic NMO lesions of the spinal cord and the optic nerves [3].

Brain involvement in NMO patients seems to be more frequent than previously described. Brain MRI at the beginning of the disease is usually normal. However, after a disease course of several years, non-MS-like lesions were found in 50% of patients, whereas MS-like lesions were present in only 10% of cases [4]. Logically, brain lesions in NMO are localized at sites of high AQP4 expression [5]. Using diffusion tensor imaging, brain tissue abnormalities have been found in normal appearing grey matter (NAGM) and normal appearing white matter (NAWM) [6]. Furthermore, using magnetization transfer (MT) MRI, Rocca et al., showed reduced MT ratio of the NAGM [7]. However, magnetic resonance (MR) spectroscopy showed no abnormality in brain NMO, including grey matter (GM) and white matter (WM) [8], [9], [10].

Only a few NMO patients have undergone brain pathology analysis [4], [11], [12]. In these cases, macrophage and eosinophil infiltration was found in brain lesions, as previously described in spinal cord lesions [11]. But no pathological data has been published on NAWM and NAGM of brain NMO patients.

We recently demonstrated that NMO patients have cognitive dysfunctions, a finding subsequently confirmed by others [13], [14]. The main cognitive deficits were found in long-term memory, speed of information processing, attention and executive functions. As far as we are aware, there have been no previous studies on brain atrophy and cognitive functions in NMO patients.

In the present study, we aimed to investigate whether there are correlations between cognitive dysfunctions and global and/or focal GM and WM volume/concentration in NMO patients.

## Materials and Methods

### Ethics Statement

All NMO patients and healthy subjects gave their written informed consent to participate in the study. The study was approved by the University Hospital of Strasbourg ethics committee.

### Setting and Participants

Twenty-eight patients with definite NMO according to the recently revised criteria [15], and 28 healthy volunteers (control group) matched for sex, age and educational level were included in the study. Patients’ and controls’ characteristics are detailed in Table 1. Patients were recruited at the Department of Neurology (University Hospital of Strasbourg). Twenty-eight patients fulfilled the revised criteria for NMO [15]. All patients had a Beck Depression Inventory score <10 [16], and an EDSS score ≤6.5 [17]. Testing for NMO-IgG was conducted using indirect immunofluorescence analysis, as previously described [18]. Eight patients, including three with cognitive impairment, had also participated in the previously published multicentre study on cognitive functions in NMO [13].

**Table 1:** Clinical and demographic features of patients with neuromyelitis optica and controls.

		Patients, n = 28	Controls, n = 28	P Value
Age a		45.3 years (11.7)	42 (15.6)	>.05
Years of education a		12.2 (2.9)	12.9 (3.4)	>.05
Female/male		19/9	19/9	>.05
EDSS score b		3.42 (0–6.5)	NA	NA
Disease duration (years) aImmunosuppressant treatment cImmunomodulatory treatment c		11.9 (10.9)152	NANANA	NANANA
First signs		Optic neuritis (n = 17), myelitis (n = 8), optic neuritis and myelitis (n = 2), brainstem signs (n = 1)	NA	NA
NMO-IgG+(%)		12 (43)	NA	NA
CSF	Protein (g/l)	0.51	NA	NA
	White cells/mm3a	27.0 (45.2)	NA	NA
	Positive IgG (%) OCB	18	NA	NA

a mean (standard deviation);

b median (range); EDSS  =  Expanded Disability Status Scale;

c At the time of cognitive tests and brain MRI. OCB  =  oligoclonal bands

NA  =  not applicable.

### Neuropsychological Assessment

We assessed NMO patients using the the BCcogSEP (*Batterie Courte d'évaluation cognitive destinée aux patients souffrant de Sclérose En Plaques*) [13], [19]. The BCcogSEP is a battery of tests to evaluate cognitive functions and was specially designed for MS patients. It is the French modified version of the Brief Repeatable Battery of Neuropsychological tests for Multiple Sclerosis (BRB-N) proposed by Rao and comprises the 5 modified BRB-N tests: a Selective Reminding Test (BCcog-SRT), a visuospatial memory test (10/36), the Paced Auditory Serial Addition Test (PASAT), a verbal fluency test, the digit symbol substitution test of the WAIS-R (DSST) [20]. The BCcog-SRT presents a list of 15 unrelated words. Upon administration of the first trial, participants are asked to recall as many words as possible. Over each of ten more trials, only the words missed on the previous trial are presented, but participants are asked to recall as many words as possible. Following a 20 minutes delay, patients are again asked to recall as many words from the list as possible. Common indices are the mean number of words recalled during the first phase (called “mean number of words”), the percentage of words systematically recalled on each trial (called “learning”), and the number of words recalled after the delay interval, or delayed recall (called “SRT-DR”).

Three tasks were added in order to provide additional information about working memory and executive functions: the crossed tapping test, a “Go-No-Go” test, the WAIS-R digit span subtest. Cognitive impairment was considered if four or more of the 14 subtests were inferior to the 5th percentile, as described by Dujardin [19].

For the four blind patients we did not perform the 10/36 and the DSST.

### Structural Image Analysis

MRI examination of participants was performed on a 1.5 Tesla whole body unit, using a standard head coil (SIEMENS Avanto MR, Erlangen, Germany). Brain MRI was performed the same day as the neuropsychological assessment.

The following sequences were acquired:

(1) 3D high resolution T1-weighted (MPRAGE) images (TR = 1900, TE = 2.68, TI = 1100; flip 15°; FOV 230 mm; matrix 260×320; slice thickness 1.0 mm; voxel size: 1×1×1 mm);
(2) Axial turbo spin-echo (TSE) T2-weighted sequence (TR = 4000, TE = 14/109, echo train length = 5, FOV 220×165 mm^2^, matrix 256×192, slice thickness 4.0 mm);
(3) Axial FLAIR T2-weighted sequence (TR = 9000, TE = 87, TI = 2500, echo train length  = 16, FOV 230 mm, matrix 224×256, slice thickness 4.0 mm ).

Global brain volume was estimated for each subject with high-resolution T1-weighted images according to the SIENAx method (Image Analysis Group, Oxford, UK) [21], [22]. Briefly, SIENAx estimates the total brain tissue volume, from a single whole-head input image, normalized for skull size. It first strips non-brain tissue, and then uses the brain and skull images to estimate the scaling between the subject’s image and standard space. It then runs tissue segmentation with partial volume estimation to estimate the volume of brain tissue (GM, WM), and applies a normalization factor to reduce head-size-related variability between subjects.

Focal brain volume loss was also analyzed and compared for patients and controls with high-resolution T1-weighted images according to the method for VBM with SPM5 (Statistical Parametric Mapping, Wellcome Department of Cognitive Neurology, London, UK) and Matlab 7.7 software (The MathWorks, MA, USA).

Briefly, this method involves an initial segmentation of T1-weighted MRI into GM and WM images in native space, followed by a normalization of the GM and WM images to GM and WM templates in stereotactic space. To improve segmentation, the normalization parameters obtained from the nonlinear normalization of the GM and WM images are re-applied to the original whole-brain MRI and the normalized whole-brain MRI is then segmented into WM and GM images in a second segmentation step. GM and WM concentration images (i.e., not modulated by the Jacobian of the transformation) were then smoothed with a 12-mm full-width half-maximum (FWHM) Gaussian kernel.

We performed voxel-level correlations of GM or WM concentration between patients and controls, and also between NMO patients with and without cognitive impairment, using a two-sample t-test, without covariates. Statistical maps were thresholded with false discovery rate (FDR) correction with *P* value <0.05 and with a minimum cluster size of 30 voxels. MNI Space Utility (MSU, http://www.ihb.spb.ru/~pet_lab/MSU/MSUMain.html), xjView software (http://www.alivelearn.net/xjview8/)and Talairach software (http://www.talairach.org/index.html) were used to create reports about cluster localization in terms of Talairach Daemon anatomical region labels.

### Data Analysis

We considered patients to have abnormal cognitive performance if their results were inferior to the 5th percentile, using the norms of Dujardin [19]. We used the residual scores of the BCcogSEP (the difference between the expected and obtained scores) for the cognitive data analysis. The expected score is known because of normative data of the BCcogSEP corrected for age, sex, and educational level [19].

We looked for correlation between cognitive dysfunction and the following parameters: disease duration, EDSS, treatments, NMO antibodies, and also global brain volume, grey matter volume, and white matter volume (normalized). For this statistical analysis, we used the Pearson test for continuous variables, and the Spearman rank correlation test for ordinal data, using SPSS 18. To correct for multiple comparisons, a *P* value ≤0.01 was considered statistically significant, a *P* value between 0.01 and 0.05 was considered as a trend, and a *P* value >0.05 was considered not significant.

The nonparametric Mann-Whitney U test was used to compare total brain, GM, and WM volumes between patients and controls (SPSS 18).

We performed voxel-level correlations between brain concentration and cognitive performance for each subtest of the BCcogSEP using residual scores with SPM5. An explicit mask was used to ensure that only brain region with decrease concentration of matter were included in the analysis. Statistical maps were thresholded without correction with *P* value <0.001 and with a minimum cluster size of 30 voxels.

## Results

The main demographical, clinical and laboratory results are summarized in Table 1. NMO was monophasic in three cases and multiphasic in the other cases. Four patients were blind. Only three patients had inflammatory lesions of the brain but not fulfilling the Barkhof’s criteria. Two of these three patients had also cognitive impairment.

### Cognitive Impairment

Fifteen NMO patients (54%) had cognitive impairment with more than four subtests of the BCcogSEP inferior to the 5th percentile. The results of the cognitive tests are summarized in Table 2. More than 30% of NMO patients had an impairment in the following tests: the PASAT 3 seconds (3s), the DSST, the BCcog-SRT and the digit span. Among the 6 patients with 10/36 immediate recall impairment, only two patients had a mild visual acuity impairment. The comparison of NMO patients with cognitive impairment to NMO patients without cognitive impairment showed no differences in terms of age, sex,

Correlations were found between BCcog-SRT learning (*P* = 0.002, rho = –0.56) and EDSS score. A trend towards a correlation was found between PASAT 2s (*P* = 0.03, rho = –0.42) PASAT 3s (*P* = 0.03, rho = –0.42), and EDSS.

**Table 2:** Number (percentage) of NMO patients with a score inferior to the 5th percentile for each cognitive subtests of the BCcogSEP.

Cognitive Tests	Number of patients with cognitive dysfunction (percentage)
Selective reminding test (SRT)	
Mean number of words	11 (39.3)
Learning	12 (42.9)
SRT-DR (delayed recall)	9 (32.1)
10/36 Spatial Recall Test*	
Immediate recall	6 (25.0)
Delayed recall	3 (12.5)
Digit Span	
Forward	7 (25.0)
Backward	9 (32.1)
Digit symbol substitution test (DSST)*	11 (45.8)
Paced Auditory Serial Addition Test (PASAT)	
2 s	7 (25.0)
3 s	11 (39.3)
Crossed tapping	3 (10.7)
Go/no-go	4 (14.3)
Fluencies	
Phonemic	8 (28.6)
Semantic	4 (14.3)
BCcogSEP battery	15 (54)

* Blind patients were not tested for these tests.

NMO = Neuromyelitis Optica; BCcogSEP = French translation of the Brief Repeatable Battery.

No correlation was found between duration of disease, treatment, NMO-IgG antibodies and, cognitive subtests and number of subtests of BCcogSEP inferior to the 5^th^ percentile.

The comparison of characteristics of NMO patients with cognitive impairment (N = 15) to NMO patients without cognitive impairment (N = 13) showed no difference in term of age (*P* = 0.26), sex (*P* = 0.36), educational level (*P* = 0.91), EDSS (*P* = 0.12) and duration of the disease (*P* = 0.14).

### Global and Focal Brain Volume

#### Global brain volume

Using SIENAx, global brain volume of NMO patients was reduced compared to controls (*P* = 0.006; Mann-Whitney U). WM brain volume of NMO patients was also reduced compared to controls (*P* = 0.005; Mann-Whitney U). No difference in GM brain volume was found between NMO patients and controls (*P* = 0.81; Mann-Whitney U). Mean whole brain volume of NMO patients was 1582244.1 mm3±92733.8 and mean whole brain volume of controls was 1645341.6 mm3±68529.5. Mean WM brain volume of NMO patients was 762744.1 mm3 ± 59667.7, and mean WM brain volume of controls was 816182.4 mm3±66658.9. Mean GM brain volume of NMO patients was 819500.1 mm3±79067.8, and mean GM brain volume of controls was 829159.2 mm3±87229.7.

Correlation was found between the number of subtests inferior to 5^th^ percentile and normalized global brain volume (*P* = 0.007, rho = –0.50). A trend towards a correlation was found between the number of subtests inferior to the 5^th^ percentile to the BCcogSEP and normalized WM volume (*P* = 0.02, rho = –0.43).

A trend towards a correlation was found between normalized WM volume and PASAT 3s (*P* = 0.024, rho = 0.40) and PASAT 2s (*P* = 0.046, rho = 0.36).

Using SIENAx, global brain volume of NMO patients with cognitive impairment (N = 15) was reduced compared to global brain volume of NMO patients without cognitive impairments (N = 13) (*P* = 0.006; Mann-Whitney U). WM brain volume of NMO patients with cognitive impairment was also reduced compared to WM volume of NMO patients without cognitive impairment (*P* = 0.005; Mann-Whitney U). No difference was found between GM brain volume of NMO with and without cognitive impairment (*P* = 0.22; Mann-Whitney U).

#### Focal brain volume

**Figure 1:**
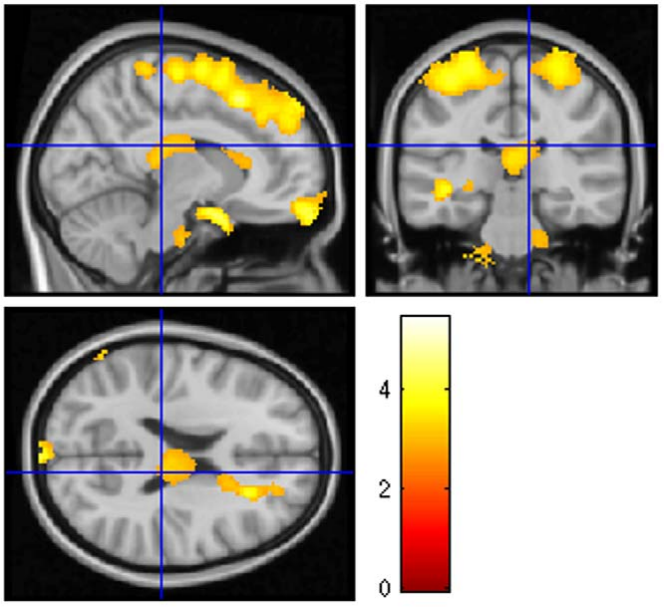
White matter volume regional differences between the NMO group (n = 28) and the control group (n = 28) using VBM. Note that the NMO group overall showed a decreased WM volume in the frontal and parietal lobes (including the superior longitudinal fascicle), corpus callosum, cerebellum, brainstem and optic chiasm compared to healthy control subjects. (*P*<0.05, false discovery rate, cluster size threshold >30 voxels).

Using VBM, focal brain atrophy of the WM was found as shown in Fig. 1 and Table 3. No focal brain atrophy of the GM was found.

**Table 3:** Clusters of significant white matter decrease in NMO patients relative to controls.

Region	BA vicinity	Side	No. of voxels	T	Coordinates (mm)			P
					x	y	z	
Frontal lobe and parietal lobes, superior frontal gyrus, precentral gyrus, postcentral gyrus and frontal superior medial	1, 2, 3, 6, 8 and 9	Bilateral	14750	5.47	–12	6	70	0.009
Frontal lobe, superior and medial frontal gyrus, frontal superior orbital	11	R	458	4.98	6	66	–26	0.010
Optic chiasm	NA	Bilateral	994	4.71	8	6	–22	0.010
Cerebellum, posterior lobe	NA	R	188	4.29	60	–46	–34	0.019
Frontal lobe, sub-gyral, anterior corpus callosum and superior longitudinal fascicle	NA	R	927	4.17	22	28	20	0.013
Cerebellum, anterior lobe, culmen of vermis	NA	L	433	4.03	–2	–66	–10	0.017
Temporal lobe, superior and middle temporal gyrus	22	L	206	3.99	–42	–28	–6	0.015
Cerebellum, posterior lobe and brainstem, pons and middle cerebellar peduncle	NA	L	663	3.83	–16	–30	–54	0.018
Frontal lobe, middle frontal gyrus	8	L	76	3.77	–40	28	46	0.019
Cerebellum, posterior lobe	NA	R	53	3.70	54	–54	–56	0.020
Posterior corpus callosum	NA	Bilateral	848	3.50	2	–26	14	0.023
Limbic lobe, hippocampus and optic tract	NA	L	104	3.47	–24	–22	–8	0.023
Temporal lobe, superior temporal gyrus and insula	38, 21	R	80	3.44	50	6	–10	0.023
Cerebellum, anterior lobe, culmen	NA	Bilateral	206	3.33	–54	–44	–26	0.026
Parietal lobe, postcentral gyrus	40	L	39	3.28	–72	–22	16	0.028
Brainstem, pons	NA	R	243	3.21	12	–16	38	0.030

For each cluster, the extension in which local maxima are located, along with the coordinates in the MNI space and the T level of the most significant voxel are reported.

T = *P*<0.05, corrected using false discovery rate (FDR). BA = Brodmann’s area, L = left, R = right, NA = not applicable.

#### Correlations between focal brain volume and cognitive tests

Because we did not find any decreased volume in GM of NMO patients, we did not look for correlations between GM volume and cognitive tests.

No correlation was found between few cognitive subtests (crossed tapping test, Go/No-Go test, and semantic fluency) and regional white matter volume in the NMO group.

Correlations were found between regional white matter volume and the PASAT 3s, DSST, phonemic fluency, forward and backward digit span, BCcog-SRT and 10/36 spatial memory test, as shown in Table 4.

**Table 4:** Correlation between performance on cognitive tests and regional white matter volume.

Cognitive subtests	Regions of white matter	BA vicinity	Side	No. of voxels	T value	x	y	z	P
10/36 imm. recall	Parietal lobe, inferior parietal lobule, supramarginal gyrus	40, 2	R	265	5.61	54	–30	28	0.000
	Parietal lobe, postcentral gyrus, inferior parietal lobule	40, 3, 2	L	140	5.20	–34	–38	58	0.000
	Parietal lobe, inferior parietal lobule, subgyral	13	L	222	5.08	–40	–40	24	0.000
	Limbic lobe, parahippocampal gyrus, hippocampus, uncus	NA	R	2475	5.02	24	–8	–26	0.000
	Frontal lobe, middle frontal gyrus, precentral gyrus	6	L	132	5.02	–44	2	44	0.000
	Cerebellum, anterior lobe, vermis	NA	R/L	458	4.62	8	−52	−34	0.000
	Limbic lobe, parahippocampal gyrus	NA	L	133	4.49	−26	−10	−28	0.000
10/36 delayed recall	Frontal lobe, middle frontal gyrus, precentral gyrus	6	L	270	6.82	−46	4	48	0.000
	Parietal lobe, inferior parietal lobule and Limbic lobe, parahippocampal gyrus, including the superior longitudinal fascicle, the corpus callosum and the chiasm	40	R/L	8537	6.39	50	−32	26	0.000
	Cerebellum, anterior lobe, nodule, vermis	NA	R	1391	5.89	8	−58	−34	0.000
	Frontal lobe, middle frontal gyrus	46	R	175	5.83	32	34	16	0.000
	Frontal lobe, medial frontal gyrus	32, 10	L	158	5.49	−18	48	−4	0.000
	Parietal lobe, postcentral gyrus	3, 40	L	576	5.47	−34	−38	58	0.000
	Occipital lobe, subgyral, inferior occipital gyrus	18	L	410	5.39	−32	-106	−12	0.000
	Frontal lobe, superior frontal gyrus, supplementary motor area	6	R	141	5.31	14	16	60	0.000
	Frontal lobe, middle frontal gyrus, middle fronto-orbital gyrus	10, 11	R	217	5.21	48	52	−8	0.000
	Occipital lobe, cuneus	17	R	260	5.11	22	-100	−6	0.000
	Occipital lobe, middle occipital gyrus	19, 18	L	193	5.06	−54	−86	−6	0.000
	Frontal lobe, middle frontal gyrus, precentral gyrus	6	R	503	4.99	44	6	54	0.000
	Temporal lobe, middle temporal gyrus	20, 37, 21	L	127	4.79	−58	−40	−18	0.000
	Frontal lobe, middle frontal gyrus	46	L	200	4.78	−54	38	32	0.000
	Parietal lobe, inferior parietal lobule	40, 2	L	190	4.42	−54	−36	34	0.000
Phonemic fluency	Thalamus	NA	L/R	430	5.79	8	−20	10	0.000
Forward digit span	Temporal lobe, middle temporal gyrus	21, 20, 37	L	155	5.10	−62	−42	−12	0.000
Backward digit span	Frontal lobe, medial frontal gyrus	25	L	33	4.14	50	8	62	0.000
DSST	Frontal lobe, middle frontal gyrus, precentral gyrus	6, 4	R	173	5.00	52	8	54	0.000
PASAT 3s	Brainstem, pons	NA	R	103	4.68	12	−18	−42	0.000
	Frontal and parietal lobes, precentral gyrus, postcentral gyrus	1, 6, 4, 3	L	252	4.48	−54	−26	64	0.000
	Frontal lobe, precentral gyrus	6	R	54	4.41	56	−2	60	0.000
	Corpus callosum	NA	R	63	4.23	4	22	10	0.000
	Frontal lobe, supplementary motor area	6	L	79	4.20	−16	−6	62	0.000
	Frontal lobe, paracentral lobule	3, 6, 4	R	96	4.03	12	−34	64	0.000
	Frontal lobe, inter-hemispheric, subcallosal gyrus	25	R/L	209	4.32	6	6	−20	0.000
	Corpus callosum	NA	R/L	251	3.97	10	−8	18	0.000
	Frontal lobe, superior frontal gyrus, supplementary motor area	6	R	98	3.96	18	12	64	0.000
SRT, mean number of words	Frontal lobe, middle frontal gyrus	6	R	36	3.95	8	−12	16	0.000
	Fornix, thalamus	NA	R	43	3.61	8	−14	18	0.000
SRT, delayed recall	Temporal lobe, subgyral, hippocampus	NA	L	35	4.06	−20	−84	0	0.000
	Occipital lobe, lingual gyrus	17	L	34	3.80	−38	−30	−6	0.000
	Fornix, thalamus	NA	R/L	95	3.59	8	−12	16	0.000

T = P<0.001, uncorrected, extent threshold of 30 voxels.

BA = Brodmann’s area, DSST = Digit Symbol Substitution Test, imm. = immediate, L = left, R = right, NA = not applicable, PASAT = Paced Auditory Serial Addition Test, SRT = Selective Reminding Test.

BCcog-SRT was correlated to the fornix, and the WM of middle frontal gyrus, hippocampus and lingual gyrus. Backward digit span was correlated to WM of the left medial frontal gyrus, and forward digit span to the left middle temporal gyrus. Phonemic fluency was correlated to the WM of thalamus.

**Figure 2:**
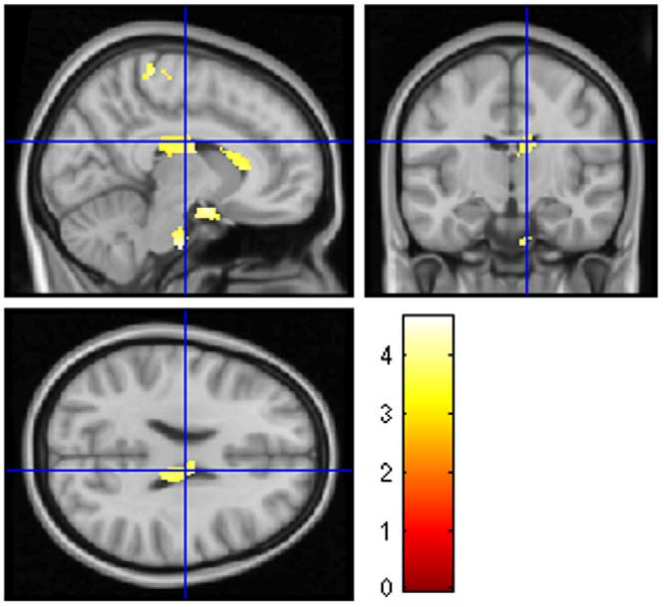
Correlation between performance on PASAT 3 seconds and regional white matter volumes of 28 NMO patients, using VBM. Note the correlations with corpus callosum and the pons. Statistical maps were thresholded without correction, with *P* value <0.001 and with a minimum cluster size of 30 voxels.

DSST was correlated to the WM of the middle frontal gyrus and precentral gyrus. PASAT 3s was correlated to the WM of corpus callosum, frontal and parietal regions and the pons (Fig. 2).

Two main structures were correlated with 10/36 immediate recall: parietal lobe and limbic lobe, including parahippocampal structures. Many white matter structures were correlated with 10/36 delayed recall: parts of the visual system (optic chiasm and occipital lobe), parts of the frontal lobe, part of the limbic system including parahippocampal regions, part of the parietal lobe including post-central gyrus and inferior parietal lobule, and the vermis.

#### Comparison of focal brain volume between NMO patients with and without cognitive impairment

**Figure 3:**
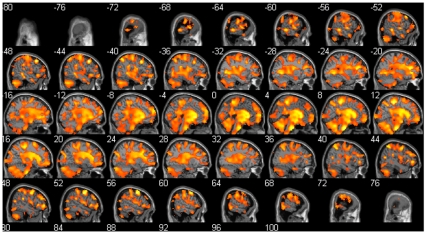
White matter volume regional differences between NMO patients with cognitive impairment (n = 15) and NMO patients without cognitive impairment (n = 13) using VBM. Note that the NMO patients with cognitive impairment have a large decreased WM volume including brainstem, cerebellum, corticospinal tracts but also the important fascicles of the brain such as corpus callosum, superior longitudinal fascicle and inferior longitudinal fascicle. (*P*<0.05, false discovery rate, cluster size threshold >30 voxels). Sagittal view of the brain using xjview.

We did not find any focal GM volume difference between NMO patients with cognitive impairment (n = 15) and without cognitive impairment. On the contrary, a large decrease of the WM when we compared NMO patients with cognitive impairment to NM patients without cognitive impairment was found (Fig. 3). These differences included brainstem, cerebellum, corticospinal tracts but also important fascicles of the brain such as corpus callosum, superior longitudinal fascicle and inferior longitudinal fascicle.

## Discussion

Our data show that 54% of NMO patients had cognitive impairment according to their BCcogSEP performances. We also found a decreased global and focal white matter volume in NMO patients compared to healthy subjects. Furthermore, we observed that the majority of the WM is decreased in NMO patients with cognitive impairment compared to NMO patients without cognitive impairment. We observed also correlations between cognitive tests performance and white matter volume. We did not find any global and focal GM differences between NMO patients and controls, and also between NMO patients with and without cognitive impairment.

We demonstrated cognitive impairment in more than half of NMO patients in a previously published multicentre study [13]. Recently, Saji et al. and He et al. also reported cognitive impairment in NMO patients [14], [23]. In these three studies and the present study, the cognitive pattern in NMO patients was a “subcortical” cognitive impairment, including a decreased speed treatment of information (DSST, PASAT), executive function impairment (PASAT, fluencies), attention impairment (PASAT, forward and backward digit span) and memory impairment (BCcog-SRT, 10/36). The term “subcortical” was first used by K. Wilson when he described in 1912 patients with Wilson’s disease with cognitive deficits different from other dementias, and then for patients with degenerative extrapyramidal disorders (Cummings, 1986). This term is also used for inflammatory diseases. This term “subcortical” is particularly coherent with NMO patients where the cognitive pattern and the atrophy pattern seem to be linked to the WM involvement.

In the previous two studies, there was no correlation between brain lesions and cognitive impairment[13], [14]. In the present study, only three patients had brain inflammatory lesions, and among them two patients had cognitive impairment.

We wanted also to better understand the relationship between white and grey matter volumes and cognitive functions in NMO patients. We did not find any decrease of brain GM volume in NMO patients, using SIENAx for global volume and VBM in SPM5 for focal volume. Moreover, the comparison of global and focal GM of NMO patients with and without cognitive impairment did not show any decreased volume. Similarly using MR spectroscopy of the brain we did not find any abnormality in the GM in an earlier study [8], and pathological analysis of brain NMO patients did not reveal cortical demyelination [24]. However, it seems that brain NAGM in NMO has a decreased MT ratio [7]. Furthermore, abnormal diffusivity (increased mean diffusivity) of NAGM has been reported [6]. These abnormalities of grey tissues have mainly been found in regions connected with the spinal white matter tracts and optic nerves [6].

We found a decrease in WM brain volume in NMO patients, using SIENAx for global volume and VBM in SPM5 for focal volume. This result, found using two different methods, strengthens the discovery of such atrophy. Abnormalities of NAWM in the brain of NMO patients were previously found using diffusion tensor imaging [6], [7]. Yu et al. found a higher average mean diffusivity (MD) and a lower average fraction of anisotropy (FA). MD measures the average molecular motion and is affected by integrity, and FA reflects the degree of alignment of cellular structures and their structural integrity [25]. Thus, subtle lesions of the WM could be responsible for abnormal diffusion and atrophy. Using VBM, we found a decrease in the WM concentration in particular regions. First, we found atrophy in the optic chiasm and optic tracts, which are regions known to be involved in NMO. We also found atrophy in regions connected to the cortico-spinal tract, such as the WM in the brainstem and the precentral and postcentral gyri. These data are coherent with the known involvement of the spinal cord and the optic nerves in NMO. One can suppose that retrograde degeneration is potentially responsible for this atrophy of white matter. Yet, we also found atrophy of other regions not involved in the optic or corticospinal tracts. Thus, we found WM atrophy of the cerebellum: this data is coherent with previous studies that have found cerebellum lesions in NMO [4], [26]. Neuropathologically, Popescu et al., did not find any demyelination of the GM of the cerebellum, and more pathological data are needed on the brain WM, even if case of cerebellum involvement has been described [24], [27]. WM atrophy of many parts of frontal, parietal (including superior longitudinal fascicle), temporal and corpus callosum regions has been found (Figure 1 and Table 3). These data would appear to conflict with the results we previously published concerning the analysis of the brain of NMO patients with MR spectroscopy, in which no abnormality was found in the WM. However, in that study we focused the analysis on the centrum ovale [8]. To our best knowledge, no study has tested the whole brain using MR spectroscopy in NMO.

We also observed correlations between cognitive test performances and WM volumes. First, we found an association between low performance for immediate and delayed spatial memory (10/36 immediate and delayed recall) and a reduced volume of the optic chiasm, the corpus callosum, limbic lobe including parahippocampal gyri and fronto-parieto-occipital regions including the superior longitudinal fascicle. Visual impairment could explain difficulties in performing a visual memory task. Thus, the correlation found with the optic chiasm is logical. Chronic disconnection of the corpus callosum by surgery is known to be responsible for moderate memory impairment, particularly topographical memory [28]. Moreover, some cases of stroke in the corpus callosum and posterior thalamus have shown memory impairment [29]. The parahippocampal gyri are regions well-known to be involved in memory, particularly in spatial memory (where stream) [30].Finally, the fronto-parietal network, including the superior longitudinal fascicle and the parietal cortex are also of importance for memory, particularly spatial working memory [31], [32].

Reduced WM in the pons was correlated with poor performances on PASAT. Norepinephrine-synthesizing neurons that send diffuse projection from a part of the pons, the locus coeruleus, have a major role in attention, particularly in focused attention and the ability to redirect attention [33], [34]. Attention is of high importance to succeed in performing the PASAT. PASAT 3s was also correlated with the corpus callosum. Anterior callosal abnormalities are reported to be correlated with impaired PASAT performance in MS [35]. In the same way, impaired PASAT was associated with numerous little frontal WM regions involved in the working memory [36]. BCcog-SRT, a verbal memory test, was found to be correlated logically to regions of importance for episodic memory (thalamus, fornix, hippocampus, frontal lobe) and for recognizing words (lingual gyrus) [37]. Digit span was found to be correlated with perisylvian atrophy, as previously described [38]. DSST, a speed writing test, was found to be correlated with the precentral gyrus, which is also the primary motor cortex, indispensable to do this test.

He et al., have recently demonstrated significantly correlations between corpus callosum, frontal regions and cognitive tests concerning verbal memory and speed of information processing [23]. Thus, with PASAT, they found correlations with corpus callosum (fraction of anisotropy and mean diffusivity).These data are coherent with ours showing also the involvement of the corpus callosum.

The diminished WM volume of NMO patients with cognitive impairment compared to NMO without cognitive impairment is highly diffuse (Figure 3). The majority of the brain WM is involved using the VBM method. Moreover, the same result is found using the SIENAx method with a high significance. Because there was no difference in terms of age, sex, educational level, NMO-IgG antibodies and EDSS, these quite clear results raise to the question: is there two types of NMO patients: those with cognitive impairment and perhaps brain involvement and those without the twos. The figure 3 illustrating this result confirms also the involvement of cerebellum, brainstem, corpus callosum, superior longitudinal fascicle and many WM regions of the brain including frontal, parietal, temporal and occipital regions, in the cognitive impairment of NMO patients.

WM atrophy detected with two methods and correlations between WM and cognitive tests, are important arguments in favour of brain WM involvement in NMO. NMO is an inflammatory disease of the CNS. AQP4 is known to be the main target antigen involved in this disease. AQP4 is a bidirectional water channel, found on all surfaces of astrocytes. The highest concentration of AQP4 is in the perivascular and peripial end-feet, in direct contact with the endothelium and pia mater, respectively. AQP4 immunoreactivity of the WM in the brain is highest and mesh-like in the chiasm, the optic nerves and the brainstem, particularly in the pons (at the floor of the fourth ventricle) [39]. These regions were found to be atrophic in NMO patients in comparison with controls. The other areas of white matter found to be atrophic, including the frontal and parietal lobes and the corpus callosum, have more limited AQP4 concentration on the surface of blood vessels [39]. This discrepancy between atrophy and AQP4 concentration in these area could be explained partly by a secondary degeneration caused by lesions in the spinal cord and optic nerves [40].

There are some limitations to our study. Firstly, our cohort of NMO patients has only 43% of patients with NMO-IgG positivity. In North American patients, particularly in the Mayo Clinic (Rochester, MN, USA), NMO-IgG positivity was reported to be 73% [2]. However, a more recent figure for NMO-IgG positivity ascertained by immunofluorescence in the Mayo clinic is 58% (95% confidence interval: 42–72) [41]. Moreover, we demonstrated that among 111 patients in a French NMO cohort, only 54% had positive NMO-IgG and this test was necessary in order to confirm the diagnosis, using Wingerchuk’s criteria, in only 10% of the cohort [15], [42]. Secondly, the SIENAx method seems to have some limitations to evaluate brain atrophy. Thus, Sharma et al., recently demonstrated, using an atrophy simulation of the brain, with various sources of error (bias-field inhomogeneity, noise, etc.), that SIENAx has a mean absolute error of evaluation of 3 to 4% (against 0.5% for SIENA) [43]. Nonetheless, we also found white matter atrophy in NMO patients when we used SPM5 software.

### Conclusion

Fifty-four percent of the NMO patients in our study had cognitive impairment according to their performance on the French version of the BRB-N. We found also a decreased global and focal white matter volume in the brain of NMO patients compared to healthy subjects. Furthermore, NMO patients with cognitive impairment have an important atrophy of brain WM compared to NMO patients without cognitive impairment. We observed also correlations between cognitive tests performances and WM concentrations. Further studies are now needed to elucidate more precisely the origin of cognitive impairment in the brain of NMO patients, particularly in the WM. We are therefore planning to use diffusion tensor imaging (DTI) to analyse the correlations between cognitive impairment and DTI parameters. More pathological data are also needed to better understand the mechanisms of cognitive impairment in NMO patients.
